# The development of the Japanese version of the compassionate engagement and action scales

**DOI:** 10.1371/journal.pone.0230875

**Published:** 2020-04-01

**Authors:** Kenichi Asano, Yasuhiro Kotera, Masao Tsuchiya, Ikuo Ishimura, Shuzhen Lin, Yuki Matsumoto, Marcela Matos, Jaskaran Basran, Paul Gilbert

**Affiliations:** 1 Department of Psychological Counseling, Mejiro University, Tokyo, Japan; 2 The Japanese Centre for Compassionate Mind Research and Training, Tokyo, Japan; 3 Online Learning, University of Derby, Derby, United Kingdom; 4 Research Department, Advantage Risk Management Company Limited, Tokyo, Japan; 5 Faculty of Applied Psychology, Tokyo Seitoku University, Tokyo, Japan; 6 Graduate School of Comprehensive Human Sciences, University of Tsukuba, Tsukuba, Japan; 7 Human Life Sciences, Tokushima Bunri University, Tokushima, Japan; 8 Center for Research in Neuropsychology and Cognitive and Behavioral Interventions (CINEICC), University of Coimbra, Coimbra, Portugal; 9 College of Health and Social Care Research Centre, University of Derby, Derby, United Kingdom; University of Florence, ITALY

## Abstract

The last few years have seen increasing research on self-report measures of compassion. The Compassionate Engagement and Action Scale (CEAS) is rooted in an evolutionary approach to compassion, which focuses on the competencies of compassion those are engagement with distress or suffering, and taking action to alleviate and prevent it. This study sought to validate the CEAS in a Japanese population using a cross-sectional design. A total of 279 students (82 males, 191 females, 6 unknown) answered self-report questionnaires, including the Japanese version of CEAS. We found single-factor structures for compassion for others scales, compassion from others scales, and compassion for self scales. All scales were found to have acceptable internal consistency, test-retest reliability, content validity, and construct validity. Even though some limitations, these results indicate that the Japanese version of CEAS is an adequately constructed and useful measure to assess compassionate engagement and action toward others, from others, and for the self with Japanese population.

## Introduction

The last 20 years have seen an upsurge of interest in prosocial behaviour and in particular compassion, with evidence that it has powerful physiological effects and aids in emotion regulation and moral behaviour [1–5]. To date there are slightly different models, definitions and measures of compassion [6, 7]. For example one scale for measuring just self-compassion is the Self-Compassion Scale (SCS). This defines self-compassion as “being open to and moved by one’s own suffering, experiencing feelings of caring and kindness toward oneself, taking an understanding, non-judgmental attitude toward one’s inadequacies and failures, and recognizing that one’s own experience is part of the common human experience” [8]. The SCS consists of six factors, which are self-kindness, self-judgement, common humanity, isolation, mindfulness, and over-identification. While these factors are of interest and are linked to mental health difficulties and well-being, the division into positive and negative factors and the use of the scale as a single score is controversial. [9–12]. Many researchers suggest the use of two factors not one [13].

Another recently developed measure is rooted in the evolutionary model of compassion, for the therapy. This is the Compassionate Engagement and Action Scales (CEAS). The CEAS is based on the idea that all motives have a stimulus response algorithm underpinning them. Since compassion evolved from mammalian parental caring behaviour the algorithm is sensitivity to the needs and suffering of the infant associated with taking action to do something about. Hence this is the root of the evolutionary approach, compassion has considerable overlaps with Buddhist approaches and supports a definition of “sensitivity to suffering in self and others with a commitment to try to alleviate and prevent it” [7].Thus, the first aspect refers to being sensitive and engaging with suffering and difficulty, and the second refers to attempting to work out how to be helpful and what to do.

Hence the scale includes items that are linked to being able to engage with distress and suffering (such as being sensitive to distress), and items for taking action to alleviate and prevent distress and suffering (such as thinking about how to be helpful). In addition, as compassion is a social process, it can be seen as a social flow in three orientations that can permeate interactions. Hence, i) there is the compassion we can feel for and direct at other people, ii) there is the compassion from others that we are aware of and respond to and iii) there is the compassion that we can bring to our own state of suffering and difficulty. Consequently the CEAS has three separate scales measuring these flows. Considering these three flows enables us to evaluate compassion from multilateral way including interactions between giving, and receiving compassion [6].

### Compassion for others

In original research on the CEAS, compassion for others has positive small correlation with positive aspect of self-compassion which is a well-known another concept related to compassion, while negative small correlation with negative aspect of self-compassion [6]. Furthermore, compassion for others has correlation with compassionate love largely, and compassionate goal mediumly [6]. In relation to indicator of mental health, compassion for others had shown no relations to depression, anxiety, and stress, but positive medium correlation with well-being in original research on the CEAS [6]. In another report, compassion for others has also been smally correlated with an individual’s personal sense of uniqueness, and subjective well-being [14]. These results are showing that compassion for others is highly or moderately related to concepts focusing on helping others, and related to mental health modestly.

From the perspective of convergent validity, compassion for others includes noticing distress signals or indicators of suffering, and empathic connection with the suffering of others [6]. Furthermore, compassion includes empathy as an engagement aspect in its theoretical model [7]. Thus, we assume that compassion for others correlates to empathy largely (r≧.5).

Additionally, not only noticing, compassion for others also requires commitment, action for alleviation of distress, and consideration for how to do it [6]. Therefore, coping strategies such as active coping, and acceptance appear that included in peripheral area, we assume that compassion for others correlates to them mediumly (.5 > r ≧ .3).

### Compassion from others

In original research on CEAS, being open and receptive to compassion from others has also small to medium correlations with positive aspect of self-compassion, self-reassure, compassionate love, and compassionate goal [6]. Additionally, compassion from others has negative small correlation with negative aspect of self-compassion, and self-criticism [6]. In relation to indicator of mental health, compassion from others has small negative correlations with depression, and stress, and positive medium correlation with well-being [6]. In other reports, compassion from others has positive small to medium correlations to early memories of warmth and security, and safe affect, and small negative correlation to depression [14]. These results demonstrate that compassion from others is related to concepts which focus on mental health or self-related concepts to a modest-moderate degree, but are not verified the relation to receiving help from others yet.

From the perspective of convergent validity, compassion from others includes experiences or feelings that people around us are supportive when we need their help [6]: it is assumed that compassion from others relates to our recognition of social support largely. Hence we assume that compassion from others correlates largely to use of emotional and instrumental support from others (r≧.5).

### Compassion for self

In original research on CEAS, compassion for self has positive large correlations with positive aspect of self-compassion, and negative small correlation with the negative aspect of self-compassion [6]. Furthermore, compassion for self has positive small to medium correlations with self-reassure, and state self-compassion, compassionate love, and compassionate goals [6]. On relation to indicator of mental health, compassion for self has negative small correlations with depression, anxiety, and stress, and positive medium correlation with well-being in original research on CEAS [6].

Previous report found that compassion for self has negative small to medium correlations with traumatic qualities of shame memories, depression, centrality of shame memories, and positive medium correlations with early memories of warmth, and positive with safe affect [15]. Another report has identified a negative medium correlation between compassion for self, and symptoms of eating disorder [16]. These results indicate that compassion for self correlates with positive aspect of self-compassion largely, and with positive concepts like well-being and compassionate love to a modest-moderate extent, and with mental health modestly. Even though compassion on the CEAS is a different concept from self-compassion, in its definition, we can see that SCS is the most relevant measurement for the CEAS at the moment. Hence, we assumed that compassion for self correlates largely to positive aspect of self-compassion (r≧.5).

Additionally, from the perspective of coping, compassion for self requires acceptance for own distress, and action for alleviation of it including plan what to do in stressed situations [6]. Thus we can consider these coping strategies are peripheral aspects of compassion for self, and assumed that compassion for self has positive medium correlations with measures of active coping, planning and acceptance (.5 > r ≧ .3). Previous research has found that self-compassion promotes personal improvement like reframing (i.e., changing a perspective of the same thing to feel differently) via acceptance [17]. Considering this report, reframing is assumed to have positive and medium correlation with compassion for self (.5 > r ≧ .3). Furthermore, when having compassion for self, people need to accept, and commit to own distress without blaming themselves. Therefore, denial, behavioural disengagement, and self-blame will be negatively correlated with compassion for self (-.5 < r ≦ -.3).

### Discriminant validity on the CEAS

From the perspective of discriminant validity, substance use is thought to be an adequate indicator for the CEAS. A previous survey has shown that alcohol use correlate to prosocial behaviour slightly which thought to be a concept reflecting behavioural aspect of compassion for others [18]. On compassion from others, some reports investigated that social support, regarded as highly related concept of compassion from others, does not correlate or correlate modestly to substance use [19] or self-reported drinking [20]. On compassion for self, a report showing that self-compassion from the SCS does not correlate to substance use [21]. Necessarily, we can regard substance or alcohol use as a good indicator of discriminant validity, and three scales of the CEAS-Japanese version (CEAS-J) are assumed to show very small correlation with substance use (r< .10).

### Aim

As mentioned above, some researches purport that the function of compassion and CEAS is a factor related to mental wellness, but each scale’s origins stem from different concepts. The CEAS, however, does not originate in Asia, and thus may not be representative of compassion in this region, at least for the scale of fears for compassion, suggesting there may a different CEAS structure be used for Asian samples [22]. Compassion is a key factor in the prediction of well-being and the prevention of mental illness, so it is meaningful to develop and examine reliability and validity of a CEAS-J.

## Materials and methods

Ethicality was maintained by following principles outlines in the Declaration of Helsinki, and the required ethics committee of Chiba University approved the study (No. 2478).

### Procedure

Participants were recruited into the study at four Japanese universities without knowledge of compassion’s psychological concept. The survey was conducted following the class of introductory psychology with paper-based questionnaire. Informed consent was provided on the face-sheet of the questionnaire, as was the purpose of the study. Written consent was not obtained in order to maintain confidentiality and anonymity of participants; instead, participants were asked to only respond to the questions posed to them if they agreed to participate in the study. After responding, participants placed questionnaire in the box. To verify the test-retest reliability, students from two universities (of the four) were recruited again in the same class. To match the data, participants were requested to answer the same four digit numbers as what they had created at the first survey. The Chiba University Ethics Committee approved the procedures.

### Participants

A total of 279 students (82 males, 191 females, 6 unknown) participated in the first survey. The mean age was 19.35 (SD = 3.26) years old. Of these, 144 (40 males, 103 females, 1 unknown) participants responded to the second survey to evaluating test-retest reliability. The mean age of the second survey participants was 19.80 (SD = 1.80) years old. About marital state, 245 students answered single, no one answered married, and 24 students were unknown (0.09%). In test-retest measurement, 137 students answered single, no one answered married, and 7 students were unknown (0.04%).

### Measurement

Measurements used were the Compassionate Engagement and Action Scales–Japanese version (CEAS-J), the Japanese version of a Self-Compassion Scale—Short Form (SCS-J-SF) [23], Depression Anxiety and Stress Scale (DASS-15) [24], the Satisfaction With Life Scale (SWLS) [25], Other-Oriented Emotional Reactivity (OOER) [26], and 10 factors from the Brief-COPE (Active coping, denial, substance use, use of emotional support, behavioural disengagement, positive reframing, planning, acceptance, and self-blame) [27]. To assess test-retest reliability, the CEAS-J was conducted twice at 3-week intervals.

In translation of CEAS, we followed preceding guidelines in order to comply with cross-cultural validity [28]. At first, the CEAS was translated into Japanese by II, a doctor of clinical psychology with translation experience, and YK, a professional psychotherapist with a masters in counselling psychology from the United States. The translation experience of II was a vital asset to this research, who has completed the Japanese translations of both compassion-focused therapy and self-compassion books in the past. After Japanese translation, the original author (KA) discussed and generated the CEAS Japanese version with its translators.

Next, the CEAS Japanese version was reviewed by an English psychologist working for a translation agency and was translated back into English. The authors (PG, JB, and MM) of both the original version of the CEAS and the Portuguese version (CEAS-P) verified the accuracy of the new version. This translation then received feedback from the previous check into consideration from the original authors, and the manuscript was translated into Japanese once again and examined for content validity. This final step was performed by KA and YM, a clinical psychologist with a PhD awarded in Australia and a Japanese teaching certification. Once the original authors of the CEAS and the Portuguese version reviewed the CEAS-J, questions and modification requests arose for back-translation. It was concluded with the authors of the Japanese version that the concerns related to differences of idiom between the languages of English and Japanese. Thus, we did not alter the Japanese version in this round.

Third, to confirm comprehensibility, 18 undergraduate students responded the CEAS-J and were instructed to address concerns about the questions in the Japanese version, and to identify any areas of difficulty in finishing the draft version. As a result, all students did not suggest amendments or raise concerns within the CEAS-J.

The CEAS-J consists of 10 items, where six assess engagement, and four assess actions of compassion on each dimension (compassion for others, compassion from others, and compassion for self). Participants were presented with a written description of compassion [29], and were asked how they would respond when confronted by their own suffering, the suffering of others, or the by being shown compassion by another, on a Likert scale of 1 (never) to 10 (always).

The SCS consists of 26 items, and which is composed of 6 subscales on a Likert scale of 1 (almost never) to 5 (almost always). As noted above, the SCS is the most popular instrument to measure self-compassion by Neff [8]. The short-form version (SCS-SF) was developed to reduce participant burden [30]. The SCS-SF has equivalent reliability and validity to the SCS, and has also been translated into Japanese [31]. We used this scale to verify convergent validity. SCS-SF consists of 12 items, and which is composed of 6 factors including three positive facets (self-kindness, common humanity, and mindfulness) and three negative facets (self-judgement, isolation, and over-identification) with two items per factor. Factor structures of the SCS and SCS-SF have been a topic of discussion [9], and because the SCS was used as two-factor structure in the development of the CEAS [6], we used the SCS as two-factor structure to determine the consistency to the original version. Participants were asked to state how often they acted in the manner stated in each of the items on a Likert scale of 1 (almost never) to 5 (almost always).

The DASS-15 is the Japanese version of the DASS-21, the condensed version of the Depression Anxiety Stress Scales [24] [32]. The DASS-21 is composed of three seven-item subscales, but the Japanese version composes only three five-item subscales due to exploratory and confirmatory factor analysis (CFA) and internal consistency ratings [24]. The DASS-15 which consists of 15 items (3 subscales are depression, anxiety, and stress) was used to confirm convergent validity in this study because the DASS-21 was used to develop the original CEAS [6]. Participants responded to Likert scales relating to their habits and experiences in the previous week, on a scale of 0 (did not apply to me at all) to 3 (applied to me very much, or most of the time). Because the CEAS has been previously found to be negatively correlated with depression in the compassion from others and compassion for self-scales, and we hypothesise that the CEAS-J would achieve a similar result to original version of the scale.

The Satisfaction With Life Scale (SWLS) is a measurement that assesses life satisfaction as a part of subjective well-being [33]. The Japanese version of the SWLS consists of 5 items and has been standardised for analytical use [25]. This scale was used to ask participants to identify the extent to which each of the items applied to them, on a Likert scale of 1 (did not apply to me at all) to 7 (applied to me very much, or most of the time). On the original version of the CEAS, the SWLS is correlated to three scales of CEAS, therefore we hypothesise that the CEAS-J would have similar results [6].

The OOER, from the Multidimensional Empathy Scale (MES), was developed to assess individual differences of empathy [26]. The MES consists of 5 factors (Other-Oriented Emotional Reactivity; OOER, Self-Oriented Emotional Reactivity, Emotional Susceptibility, Perspective Taking, and Fantasy). We used the OOER (5 items; examples are “If I saw someone crying, I would want to comfort them”, “If I found someone in trouble, I would hope that the problem resolved”) to assess construct validity of compassion for others scale. Participants were asked to answer to what extent each of the items applied to them on a Likert scale of 1 (did not apply to me at all) to 5 (applied to me very much). As aforementioned, OOER is used as an indicator of convergent validity of compassion for others.

The Brief-COPE is a questionnaire used to evaluate coping style and has been translated into Japanese [27] [34]. Ten of 14 factors from the Brief-COPE were used as factors in accordance with hypotheses. Two items were included in each factor. Participants were asked using a Likert scale of 1 (never) to 4 (always) to determine how often they engage in items relating to the ten factors. Active coping reflects the tendency to cope proactively, so for the purposes of this study, it will be analysed in relation to compassion for others and compassion for self, as they are considered to face with own or other’s sufferings. Denial reflects the tendency not to acknowledge nor accept a problem or suffering, so for this study, it will be analysed to identify a relation to compassion for self, which is considered that to face with own or other’s sufferings. Use of emotional support reflects the tendency to receive support from others for emotional issues and use of instrumental support reflects the tendency to receive active assistance in a matter. Therefore, they will be analysed in order to define a relationship with compassion from others scale. Behavioural disengagement reflects the tendency to resign or distancing oneself from problems and will be analysed alongside compassion for self. Positive reframing reflects the tendency to shift thoughts in a more optimistic light. Therefore, it will be assessed for its relation to compassion for self. Planning reflects to ability to develop plans to cope with problems and compassion for self will be analysed for a relationship. The factor of acceptance reflects the tendency of accept problems or situations and will be assessed alongside compassion for others and for self to describe a relationship. Self-blame identifies the tendency to blame or criticise oneself, and will be assessed with compassion for self to observe the relationship.

Substance use reflects the tendency to use alcohol or drug to distract from problems; thus, it will be used as indicator of discriminant validity for three scales.

Three-week test-retest reliability was analysed using the Global Rating of Change (GRC) assessment recommended in guideline [35]. On a Likert scale of 1 (improved very much) to 7 (deteriorated very much), participants identified their rate of change both physiologically and psychologically. Individuals scoring themselves between 3 and 5 were not considered ‘changed’ and were used to calculate test-retest reliability.

### Data analysis

Analysis was conducted using R version 3.2.3 [36]. Missing data on all continuous scales were handled using multiple imputation assuming missing at random.

#### Factor structure and reliability

We conducted minimum average partial (MAP) which helps to determine the number of factors objectively [37]. Next, we conducted exploratory factor analyses (EFA) assuming the factor structures proposed by MAP. In EFA, we removed items with factor loadings lower than 0.40.

To evaluate internal consistency, the scores of Cronbach’s αwere calculated. In test-retest reliability, we excluded participants with a change in GRC; only data from people who scored from 3 to 5 (regarded as ‘no change’) were analysed. After that, intra-class correlations were calculated.

#### Construct validity

To evaluate convergent, and discriminant validity, Pearson’s correlation coefficient was calculated. We interpreted the effect size r based on previous guidelines, ≧.10 as small, ≧.30 as medium, and ≧.50 as large [38].

We hypothesised that compassion for others has large correlation with OOER (r ≧ .50), and medium correlation with active coping, and acceptance (.30 ≦ r < .50). For compassion from others, it has large correlation with use of emotional support, and instrumental support (r ≧ .50). For compassion for self, it has large correlation with positive aspect of SCS (r ≧ .50), and medium with active coping, positive reframing, planning, acceptance positively (.30 ≦ r < .50), and denial, behavioural disengagement, and self-blame (-.30 ≧ r > -.50).

### Relations to mental health

Following to original version, relations to mental health (depression, anxiety, stress, and satisfaction with life) were verified by using Pearson’s correlation coefficient.

## Results

About missing value, nine students out of 279 students were incomplete (0.03%). In test-retest measurement, seven students out of 144 students were incomplete (0.04%). All of missing values were imputed using multiple imputation as mentioned in data analysis section.

### Factor structure and reliability

The results of MAP suggested a single factor structures on three scales with the smallest average squared correlation of .326, .325, .0411 in compassion for others, .325, .518, .676 in compassion from others, and .411, .521, .620 in compassion for self. Next, we conducted EFA with maximum likelihood method on each scales with single factor structures.

On compassion for others scale, an item (“I am emotionally moved by expressions of distress in others”) showed low factor loading (less than .40) and removed. The final factor structure revealed by the exploratory factor analysis, as well as the mean, standard deviation, skewness, and kurtosis of each item, are shown in Table 1. The proportion of variance explained was 61.76% and Cronbach’s α was .92.

**Table 1:** Factor loadings for the compassion for others scales.

No	Item	Factor	Mean	*SD*	skewness	kurtosis
Compassion for Others -Engagement- (*α* = .92)						
10	I think about and come up with helpful ways for them to cope with their distress.	**.876**	7.09	1.76	-0.58	0.41
6	I reflect on and make sense of other people’s distress.	**.845**	7.30	1.79	-0.70	0.49
9	I direct attention to what is likely to be helpful to others.	**.824**	6.88	1.72	-0.46	0.60
1	I am motivated to engage and work with other peoples’ distress when it arises.	**.807**	6.66	1.86	-0.37	0.01
12	I take the actions and do the things that will be helpful to others.	**.771**	6.76	1.73	-0.38	0.30
13	I express feelings of support, helpfulness and encouragement to others	**.733**	7.25	2.02	-0.84	0.46
5	I tolerate the various feelings that are part of other people’s distress.	**.731**	6.32	1.89	-0.39	-0.03
2	I notice and am sensitive to distress in others when it arises.	**.698**	6.55	1.84	-0.47	0.08
8	I am accepting, non-critical and non-judgemental of others people’s distress.	**.444**	6.62	2.05	-0.27	-0.30
	contribution (%) Text for question items reproduced from [6]	61.76				

On compassion from others scale, all items showed sufficient loadings with single factor. The final factor structure revealed by the exploratory factor analysis, as well as the mean, standard deviation, skewness, and kurtosis of each item, are shown in Table 2. The proportion of variance explained was 55.74% and Cronbach’s α was .90.

**Table 2:** Factor loadings for the compassion from others scales.

No	Item	Factor	Mean	*SD*	skewness	kurtosis
Compassion from Others -Engagement- (*α* = .90)						
10	Others think about and come up with helpful ways for me to cope with my distress.	**.903**	6.17	1.82	-0.41	-0.03
9	Others direct their attention to what is likely to be helpful to me.	**.883**	6.11	1.78	-0.53	0.14
12	Others take the actions and do the things that will be helpful to me.	**.838**	6.10	1.78	-0.21	0.11
13	Others treat me with feelings of support, helpfulness and encouragement.	**.793**	6.65	1.94	-0.53	0.31
6	Others reflect on and make sense of my feelings of distress.	**.721**	6.23	1.97	-0.58	0.19
5	Others tolerate my various feelings that are part of my distress.	**.711**	5.72	1.94	-0.21	0.12
1	Other people are actively motivated to engage and work with my distress when it arises.	**.703**	6.31	1.81	-0.51	0.65
2	Others notice and are sensitive to my distressed feelings when they arise in me.	**.532**	5.82	2.05	-0.24	-0.57
8	Others are accepting, non-critical and non-judgemental of my feelings of distress.	**.456**	5.68	1.98	-0.13	-0.33
4	Others are emotionally moved by my distressed feelings.	**.422**	4.68	2.03	0.09	-0.51
	contribution (%) Text for question items reproduced from [6]	55.74				

On compassion for self scale, three items were deleted because of low factor loadings (less than .40). They were “I am accepting, non-critical and non-judgemental of my feelings of distress”, “I am emotionally moved by my distressed feelings or situations”, and “I notice, and am sensitive to my distressed feelings when they arise in me”. The final factor structure revealed by the exploratory factor analysis, as well as the mean, standard deviation, skewness, and kurtosis of each item, are shown in Table 3. The proportion of variance explained was 50.85% and Cronbach’s α was .84.

**Table 3:** Factor loadings of in the self compassion scales.

No	Item	Factor 1	Mean	*SD*	skewness	Kurtosis
Compassion for Self (*α* = .84)						
10	I think about and come up with helpful ways to cope with my distress.	**.829**	6.63	1.86	-0.64	0.31
9	I direct my attention to what is likely to be helpful to me.	**.758**	6.41	1.96	-0.54	0.05
12	I take the actions and do the things that will be helpful to me.	**.730**	6.41	1.85	-0.60	0.26
13	I create inner feelings of support, helpfulness and encouragement.	**.671**	6.09	2.05	-0.40	-0.18
5	I tolerate the various feelings that are part of my distress.	**.551**	6.09	1.85	-0.11	-0.27
1	I am motivated to engage and work with my distress when it arises.	**.482**	6.10	2.02	-0.31	-0.17
6	I reflect on and make sense of my feelings of distress.	**.478**	6.64	1.92	-0.36	-0.11
	contribution (%) Text for question items reproduced from [6]	50.85				

Descriptive statistics and Cronbach’s *α* of all variables are shown in Table 4. The correlations (r) between three scales were from .21 to .37.

**Table 4:** Descriptive statistics and Cronbach’s α of all variables.

	Mean	*SD*	*α*
Compassion for Others	61.42	12.92	.92
Compassion from Others	59.46	13.99	.90
Compassion for Self	44.38	9.59	.84
Self-Compassion Scale Short Form			
Positive	18.27	3.93	.67
Negative	21.90	5.46	.86
DASS-15			
Depression	4.05	3.64	.84
Anxiety	2.53	3.17	.83
Stress	5.62	3.86	.81
Satisfaction With Life Scale	16.35	6.18	.85
Others Oriented Empathy Reactivity	19.19	3.45	.79
Brief COPE			
Active Coping	5.72	1.11	.46
Denial	3.07	1.29	.69
Use of Emotional Support	5.48	1.78	.85
Use of Instrumental Support	5.63	1.76	.88
Behavioral Disengagement	4.03	1.45	.79
Positive Reframing	4.86	1.45	.56
Planning	5.84	1.35	.66
Acceptance	5.92	1.25	.68
Self-Blame	5.54	1.86	.85
Substance Use	3.15	1.63	.90

To evaluate test-retest reliability, intraclass correlations were calculated for participants whose answers were in the accepted level of 3 to 5 in the GRC. One hundred forty-four of 163 participants answered in this range, and the mean age was 19.80 (SD = 1.80). Intraclass correlations of compassion for others scale were .67 (0.57–0.76); for compassion from others scale, correlations were .72 (0.63–0.79); and for compassion for self scale, correlations were .68 (0.58–0.76).

### Construct validity

Correlations of all variables are shown in Table 5.

**Table 5:** The correlations between factors of the compassionate engagement and action scales and variables.

Measures (*N* = 279)	Compassion for Others		Compassion from Others		Compassion for Self	
Compassion for Others	-		.34	**	.37	**
Compassion from Others	.34	**	-		.21	**
Compassion for Self	.37	**	.21	**	-	
Self-Compassion Scale Short Form						
Positive	.03		.13	*	.40	**
Negative	-.18	**	.11		.14	*
DASS-15						
Depression	.05		-.21	**	-.30	**
Anxiety	.11		-.14	*	-.19	**
Stress	.11		-.09		-.15	*
Satisfaction With Life Scale	.02		.27	**	.26	**
Others Oriented Emotional Reactivity	.67	**	.27	**	.28	**
Brief COPE						
Active Coping	.32	**	.15	*	.40	**
Denial	-.14	*	.01		-.30	**
Use of Emotional Support	.24	**	.51	**	.12	*
Use of Instrumental Support	.16	**	.50	**	.07	
Behavioral Disengagement	-.17	**	.01		-.33	**
Positive Reframing	.17	**	.19	**	.35	**
Planning	.25	**	.08		.43	**
Acceptance	.33	**	.03		.33	**
Self-Blame	.18	**	-.10		-.15	*
Substance Use	-.01		-.02		-.03	

*p < 0.05

** p < 0.01.

About convergent validity, compassion for others showed large correlation with OOER (r = .67), and medium correlations with active coping (r = .32), and acceptance (r = .33). Compassion from others and use of emotional support (r = .51), and use of instrumental support (r = .50). Compassion for self showed medium correlation with positive aspect of self-compassion (r = .40), active coping (r = .40), denial (r = -.30), behavioural disengagement (r = -.33), positive reframing (r = .35), planning (r = .43), and acceptance (r = .33). Lastly, compassion for self correlated with self-blame (r = -.15) modestly.

### Relations to mental health

On mental health, compassion for others did not correlate with depression, anxiety, stress, nor SWSL. Compassion from others correlated to depression (r = -.21), anxiety (r = -.14), and SWSL (r = .27) modestly. Compassion for self correlated with depression (r = -.30) mediumly, and anxiety (r = -.19), stress (r = -.15), and SWSL (r = .26) modestly.

## Discussion

### Content validity

This study sought to develop a Japanese version of the CEAS. As an accepted recommendation of Beaton and colleagues, translation procedures used two translations independent of one another [28]. Scales were reviewed by the developers of the CEAS and CEAS-P, to verify content validity, and a primary survey for students was conducted to verify comprehensibility. Because no items arose for issues or difficulties in either assessment, it is suggested that the CEAS-J has sufficient content validity and comprehensibility.

### Factor structure and reliability

All scales were suggested single-factor structure by MAP, and some items deleted on compassion for others, and compassion for self scales. Although the identification of compassion on the CEAS includes sensitivity and commitment, which were shown as engagement and action subscales separately, they were integrated into higher-order factor in the original scale [6]. A report using the CEAS for general population has also shown engagement and action items were integrated into same factor (however, the report conducted EFA including all of three scales together) [39]. Also in clinical works, compassion focused therapy or compassionate mind training develop overall compassion not dividing into engagement and action [7]. Thus, results of MAP and EFA are understandable, and it might be better to consider compassion as comprehensive concept including engagement and action in self-report measurement.

Additionally in compassion for others scale, an item which is“I am emotionally moved by expressions of distress in others” was removed because of low factor loading. Similar item in compassion for self scale (“I am emotionally moved by my distressed feelings or situations”) is also removed as with another item on compassion for self (“I notice, and am sensitive to my distressed feelings when they arise in me”). These items seemed to be describing own emotional response directly, and might be caused by the cultural differences in attitude toward own emotional response. In Eastern countries, low arousal emotions are valued more than high arousal emotions [40], and also Asian people, in general, tend to suppress own emotion than Western people [41]. Thus, being emotionally moved means upsetting or weakness rather than compassion or sensitivity on compassion in Japanese culture, and it has different function in Western cultures.

Next, an item showing non-critical attitude (“I am accepting, non-critical and non-judgemental of my feelings of distress”) was removed on compassion for self. Previous research shows that self-criticism is higher in Japanese individuals than individuals in other twelve countries [42]. Earlier research has also pointed out that self-criticism in Japanese individuals serve different functions than those in American, which might be caused by cultural differences [43]. The experimental research pointed out that Japanese self-criticism tends to operate for the purpose of self-improvement by accepting own problem or weakness [44]. Such differences on the function of self-criticism are also shown in correlations between self-criticism and other positive subscales in SCS. In U.S. sample, self-judgement showed larger correlation with positive subscales of self-compassion than Japanese sample (self-kindness; r = -.81 in U.S., r = .-.25 in Japan, common humanity; r = -.46 in U.S., r = .-.13 in Japan, and mindfulness; r = -.67 in U.S., r = .-.27 in Japan)[8][23]. These results enable us to assume that being non-critical and non-judgemental might not necessarily contribute to being compassionate in Japanese population. The function of self-criticism on compassion in Japanese population should be investigated in future studies.

The internal consistencies on the original version of CEAS were from 72. to .94 and were from 84. to .92 in the CEAS-J, indicating that the CEAS-J has enough internal consistency as original version. Intra-class correlations of test-retest reliability were found to be sufficient (from .67 to .72.) in a three-week interval, and it is believed that the CEAS-J has acceptable test-retest reliability.

### Construct validity

Most of hypotheses were supported on construct validity except for positive aspect of SCS, and self-blame on compassion for self.

Compassion for others showed large correlation with OOER, and medium correlation with active coping, and acceptance. Compassion from others showed large correlation with use of emotional support, and use of instrumental support. Thus we can conclude that these scales have enough construct validity.

Meanwhile, compassion for self showed lower correlation with positive aspect of SCS and self-blame, while it showed enough correlations with active coping, denial, behavioural disengagement, positive reframing, planning, and acceptance. In relation to positive aspect of SCS, we can assume that smaller effect size might be caused by using the short-form version of SCS[30][31]. We need to verify the reproducibility by using the original long-form version. On self-blame, we can assume that absence of non-criticism related item had caused smaller correlation. Additionally as mentioned above, we need to elucidate the relationship between compassion and self-criticism in Japanese population considering the cultural differences. However, we should conclude that compassion for self scale does not have enough convergent validity on positive aspect of SCS, yet enough for copings like active coping, denial, behavioural disengagement, positive reframing, planning, and acceptance.

### Relation to mental health

In relation to mental health, compassion for others showed correlation with satisfaction with life, but no relation to depression, anxiety, and stress in the original version [6]. However in this study, compassion for others showed small correlation with anxiety, and stress, but not relation to satisfaction with life. The relation to DASS differed only slightly, but compassion for others did not show relation to satisfaction with life. This result speculates that having compassion for others in Japanese population may include submissive function. A report introducing submissive aspects of compassion proposed that existence of compassion is a submissive behaviour [45]. A cross-cultural study showed that Japanese people have subordinate tendency than U.S. people on both of self-report and neural indicator [46]. If compassion for others in Japanese population had submissive aspect, it is reasonable to show lower effect size on satisfaction with life. Furthermore, submissive behaviour is considered as a strategy employed to avoid rejection, and it is hypothesised to be rooted in past experiences in clinical situation [45]; it is meaningful to investigate the relationship between compassion for others and submissive compassion in Japanese sample.

Compassion from others showed small correlation with depression and stress, and medium with satisfaction with life [6]. This result mostly agreed with the result in this study, we can regard the compassion from others scale has sufficient similarity to the original version.

About compassion for self, it showed small correlation with depression, anxiety, and stress, and medium with satisfaction with life in the original research [6]. Similar result was demonstrated in relation to DASS largely, however, compassion for self correlated to satisfaction with life to a small degree. The deletion of items might affect to correlation between compassion for self and satisfaction with life again. Another possibility relates to cultural difference. Previous research reported a lower level of life satisfaction in Japanese population [47] [48], and a review on cross-culture identified a difference in perception of happiness between European-American cultures and East Asian cultures [49]. In line with these findings, the compassion for self scale in this study demonstrated limited similarity to the original version in the area of satisfaction with life.

### Strengths and limitations

The main strength of this study was the ability to successfully develop a Japanese version of the CEAS. The CEAS-J can be considered a useful measurement to reveal the functions of compassion from the evolutional perspective. As mentioned earlier, there are several discussions about the specific functions of compassion and self-compassion, therefore by revealing the relationships among compassion, self-criticism, and mental well-being, more meaningful treatment can be developed to help preventing mental illness in Japanese society.

In compassion focused therapy, when patients have difficulty developing compassion for self, therapists often work with the client on understanding compassion for and from others [50]. On that account, the CEAS-J will be a useful measurement to better understand clinical work relating to Compassion Focused Therapy. However, therapists need to pay attention to possible cultural differences in compassion, and self-criticism suggested in this survey when they deliver therapy to Japanese individuals. The therapists need to be aware of clients’ self-criticism, and reflect its positive functions for the client carefully, aiming to develop client’s compassion. In addition, compassion motivates us to help others or ourselves, and it can help us address problems and understand how to alleviate or prevent them. Such motivation can be thought as a common factor on psychotherapy, so the CEAS-J will be useful for other psychotherapies as well.

There are limitations to this study. We sampled only 279 Japanese undergraduate students since in the development of the CEAS, even though undergraduate students were recruited from three countries in the original study (U.K., Portuguese, and U.S.A.) [6]. However, as we hope to reveal the functions of compassion in Japanese population, it is important to include other demographics (e.g., age, career phase, socioeconomical classes). It is recommended that the development of scales be amended to compare a more representative sample of the population, possibly by including clinical and general populations. It is also necessary to consider the factor constructions which are different from the original version. We need to test the reproducibility by using both of exploratory and confirmatory factor analyses. Last, in construct validity, some measurements scored low Cronbach’s *α*: we need to consider it in interpretation.

## Conclusion

In this study, we developed the CEAS-J based on guidelines to ensure reliability and validity by the authors of the CEAS. The results indicated different factor structure to its original version. The tests of internal consistency, test-retest reliability proved sufficient. Though some relations differed from original versions, construct validities were acceptable, and the relations between variables were identified. We can conclude that the CEAS-J has enough reliability and validity, and future studies need to reveal more detail about reproducibility and the functions of compassion with other populations.

## Supporting information

S1 File The compassionate engagement and action scales Japanese version. (DOCX)

S2 File The Dev. of CEAS-J data. (XLSX)

## References

[1] Böckler A, Tusche A, Singer T. The Structure of Human Prosociality: Differentiating Altruistically Motivated, Norm Motivated, Strategically Motivated, and Self-Reported Prosocial Behavior. Soc. Psychol. Personal. Sci. 2016; 7, 530–541.

[2] Crocker J, Canevello A. Creating and undermining social support in communal relationships: The role of compassionate and self-image goals. J. Pers. Soc. Psychol. 2008; 95, 555–575. 10.1037/0022-3514.95.3.555 18729694

[3] Davidson RJ, Harrington A. Visions of CompassionWestern Scientists and Tibetan Buddhists Examine Human Nature. Oxford: Oxford University Press; 2002.

[4] Gilbert P. Introducing compassion-focused therapy. Adv. Psychiatr. Treat. 2009; 15, 199–208.

[5] Strauss C, Lever TB, Gu J, Kuyken W, Baer R, Jones F, et al What is compassion and how can we measure it? A review of definitions and measures. Clin. Psychol. Rev. 2016; 47, 15–27. 10.1016/j.cpr.2016.05.004 27267346

[6] Gilbert P, Catarino F, Duarte C, Matos M, Kolts R, Stubbs J, et al The development of compassionate engagement and action scales for self and others. J. Compassionate Health Care. 2017; 4, 4.

[7] Gilbert P. editor. Compassion: Concepts, research and applications. Oxford: Taylor & Francis; 2017.

[8] Neff KD. The development and validation of a scale to measure self-compassion. Self and Identity. 2003; 2, 223–250.

[9] Muris P, Petrocchi N. Protection or vulnerability? A meta-analysis of the relations between the positive and negative components of self-compassion and psychopathology. Clin Psychol Psychother. 2005; 24, 373–383. 10.1002/cpp.2005 26891943

[10] Neff KD. The self-compassion scale is a valid and theoretically coherent measure of self-compassion. Mindfulness. 2016; 7, 264–274.

[11] Neff KD, Whittaker TA, Karl A. Examining the factor structure of the self-compassion scale in four distinct populations: Is the use of a total scale score justified? Journal of Personality Assessment. 2017; 99, 596–607. 10.1080/00223891.2016.1269334 28140679

[12] Neff KD, Tóth-Király I, Yarnell LM, Arimitsu K, Castilho P, Ghorbani N, et al Examining the factor structure of the Self-Compassion Scale in 20 diverse samples: Support for use of a total score and six subscale scores. Psychol Assess. 2019; 31, 27–45. 10.1037/pas0000629 30124303

[13] Brenner RE, Heath PJ, Vogel DL, Credé M. Two is more valid than one: Examining the factor structure of the Self-Compassion Scale (SCS). Journal of counseling psychology. 2017; 64, 696–707. 10.1037/cou0000211 28358523

[14] Demir M, Haynes A, Sanchez M, Parada JC. Personal sense of uniqueness mediates the relationship between compassion for others and subjective well-being. Journal of Happiness Studies. 2019; 20, 1751–1773.

[15] Steindl SR, Matos M, Creed AK. Early shame and safeness memories, and later depressive symptoms and safe affect: The mediating role of self-compassion. Current Psychology; 2018; 1–11.

[16] de Carvalho Barreto M, Ferreira C, Marta-Simões J, Mendes AL. Exploring the paths between self-compassionate attributes and actions, body compassion and disordered eating. Eat Weight Disord—Stud Anorexia, Bulim Obes. 2018; 1–7. 10.1007/s40519-018-0581-3 30229409

[17] Zhang JW, Chen S. Self-compassion promotes personal improvement from regret experiences via acceptance. Personality and Social Psychology Bulletin. 2016; 42, 244–258. 10.1177/0146167215623271 26791595

[18] Gabriella I, Sneed CD. Prosocial behavior and alcohol use among college students: A correlational study. Poster session presented at the annual meeting of the Western Psychological Association; 2018 Apr 26–29; Portland, USA. [cited 2019 Dec 19]. Available from: https://osf.io/2m7x3/wiki/home/

[19] Chou JL, Pierce KJ, Pennington LB, Seiler R, Michael J, Mc Namara D, et al Social Support, Family Empowerment, Substance Use, and Perceived Parenting Competency during Pregnancy for Women with Substance Use Disorders. Substance Use & Misuse. 2018; 53, 2250–2256. 29757060 10.1080/10826084.2018.1467456

[20] Fondacaro MR, Heller K. Social support factors and drinking among college student males. J Youth Adolescence; 1983; 12, 285–299. 10.1007/BF02088727 24306308

[21] Hayes JA, Lockard AJ, Janis RA, Locke BD. Construct validity of the Self-Compassion Scale-Short Form among psychotherapy clients. Counselling Psychology Quarterly. 2016; 29, 405–422.

[22] Asano K, Tsuchiya M, Ishimura I, Lin S, Matsumoto Y, Miyata H, et al The development of fears of compassion scale Japanese version. PLoS One. 2017; 12, e0185574 10.1371/journal.pone.0185574 29023461 PMC5638239

[23] Arimitsu K. Development and validation of the Japanese version of the Self-Compassion Scale. Japanese J Psychol. 2014; 85, 50–59. 10.4992/jjpsy.85.50 24804430

[24] Suzuki Y, Kino K. Development of the Multidimensional Empathy Scale (MES): Focusing on the distinction between self- and other-orientation. Japanese J Educ Psychol. 2008; 56, 487–497.

[25] Adachi K, Ueno T. The standardization of DASS Japanese version (Ⅰ). The 24th congress of the Japanese Association of health psychology. Tokyo; 2011. pp. 11–12.

[26] Otsuka Y. The COPE inventory: a theoretically based coping questionnaire. Hiroshima Psychol Res. 2008; 8, 121–128.

[27] Sumino Z. Jinseinitaisuru Manzokukanshakudo (the Satisfaction With Life Scale [SWLS]) nihongobansakuseinokokoromi. Annu Conv Japanese Assoc Educ Psychol. 1994; 36, 192.

[28] Beaton DE, Bombardier C, Guillemin F, Ferraz MB. Guidelines for the process of cross-cultural adaptation of self-report measures. Spine. 2000; 25, 3186–3191. 10.1097/00007632-200012150-00014 11124735

[29] The Compassionate Mind Foundation (2016) The Compassionate Engagement and Action Scales. Available: https://www.compassionatemind.co.uk/uploads/files/the-compassionate-engagement-and-action-scales.pdf. Accessed 22 December 2019.

[30] Raes F, Pommier E, Neff KD, Van Gucht D. Construction and factorial validation of a short form of the Self-Compassion Scale. Clin Psychol Psychother. 2010; 18, 250–255. 10.1002/cpp.702 21584907

[31] Arimitsu K, Aoki Y, Furukita M, Tada A, Togashi R. Construction and validation of a short form of the Japanese version of the Self-Compassion Scale. Komazawa Annu Reports Psychol. 2016; 18, 1–9.

[32] Antony MM, Bieling PJ, Cox BJ, Enns MW, Swinson RP. Psychometric properties of the 42-item and 21-item versions of the Depression Anxiety Stress Scales in clinical groups and a community sample. Psychol Assess. 1998; 10, 176–181.

[33] Diener E, Emmons RA, Larsen RJ, Griffin S. The Satisfaction With Life Scale. J Pers Assess. 1985; 49, 71–75. 10.1207/s15327752jpa4901_13 16367493

[34] Carver CS, Scheier MF, Weintraub JK. Assessing coping strategies: A theoretically based approach. J Pers Soc Psychol. 1989; 56, 267–283. 10.1037//0022-3514.56.2.267 2926629

[35] Mokkink LB, Terwee CB, Knol DL, Stratford PW, Alonso J, Patrick DL, et al The COSMIN checklist for evaluating the methodological quality of studies on measurement properties: A clarification of its content. BMC Med Res Methodol. 2010; 10, 22 10.1186/1471-2288-10-22 20298572 PMC2848183

[36] R Development Core Team. R: A language and environment for statistical computing. R Foundation by Statistical Computing; 2011.

[37] Velicer WF. Determining the number of components from the matrix of partial correlations. Psychometrika. 1976; 41, 321–327.

[38] Cohen J. Statistical power analysis for the behavioral sciences (2nd ed.). Hillsdale, NJ: Erlbaum; 1988.

[39] Lindsey S. Examining the psychometric properties of the compassionate engagement and action scales in the general population. PhD Thesis, University of Essex, 2017.

[40] Lim N. Cultural differences in emotion: differences in emotional arousal level between the East and the West. Integrative medicine research. 2016; 5, 105–109. 10.1016/j.imr.2016.03.004 28462104 PMC5381435

[41] Butler EA, Lee TL, Gross JJ. Emotion regulation and culture: Are the social consequences of emotion suppression culture-specific?. Emotion. 2017; 7, 30. 10.1037/1528-3542.7.1.30 17352561

[42] Halamová J, Kanovský M, Gilbert P, Troop NA, Zuroff DC, Petrocchi N, et al Multiple Group IRT Measurement Invariance Analysis of the Forms of Self-Criticising/Attacking and Self-Reassuring Scale in Thirteen International Samples. Journal of Rational-Emotive & Cognitive-Behavior Therapy. 2019; 1–34.

[43] Kitayama S, Markus HR, Matsumoto H, Norasakkunkit V. Individual and collective processes in the construction of the self: Self-enhancement in the United States and self-criticism in Japan. J Pers Soc Psychol. 1997; 72, 1245–1267. 10.1037//0022-3514.72.6.1245 9177018

[44] Karasawa M. A Japanese perception of self and others: Self-critical and other-enhancing biases. Shinrigaku kenkyu: The Japanese journal of psychology. 2001; 72, 195–203. 10.4992/jjpsy.72.195 11697273

[45] Catarino F, Gilbert P, Mcewan K, Baião R. Compassion motivations: Distinguishing submissive compassion from genuine compassion and its association with shame, submissive behavior, depression, anxiety and stress. J Soc Clin Psychol. 2014; 33, 399.

[46] Freeman JB, Rule NO, Adams RB Jr, Ambady N. Culture shapes a mesolimbic response to signals of dominance and subordination that associates with behavior. Neuroimage. 2009; 47, 353–359. 10.1016/j.neuroimage.2009.04.038 19376242

[47] Koyasu M, Kusumi T, Carvalho Filho MK, Hashimoto K, Fujita K, Suzuki S, et al A cross-national study on happiness: Data from thirteen countries. Japanese Psychological Review. 2012; 55, 70–89.

[48] Diener E, Diener M, Diener C. Factors Predicting the Subjective Well-Being of Nations In: Diener E., editors. Culture and Well-Being. Social Indicators Research Series, vol 38 Springer, Dordrecht; 2009.

[49] Uchida Y, Norasakkunkit V, Kitayama S. Cultural constructions of happiness: theory and emprical evidence. Journal of happiness studies. 2004; 5, 223–239.

[50] Gilbert P. Compassion focused therapy: Distinctive features. New York: Routledge; 2010.

