# Supplementary material for: The development of the Japanese version of the compassionate engagement and action scales
Source: PLoS One. 2020 Apr 1;15(4):e0230875. doi: 10.1371/journal.pone.0230875 (PMC7112184; doi:10.1371/journal.pone.0230875)
Supplement: S1 File — (DOCX) [file pone.0230875.s001.docx]

**The Compassionate Engagement and Action Scales Japanese version –** Translated from [6]

**問1**

**問2**

**問3**
